# Editorial: Digital health quality, acceptability, and cost: steps to effective continuity of cancer care

**DOI:** 10.3389/fdgth.2023.1264638

**Published:** 2023-08-11

**Authors:** Anna C. Singleton, Tanie Estapé, Carolyn Ee, Karice K. Hyun, Stephanie R. Partridge

**Affiliations:** ^1^ Engagement and Co-Design Research Hub, Faculty of Medicine and Health, The University of Sydney, Sydney, NSW, Australia; ^2^ Department of Psychosocial Oncology, FEFOC Foundation, Barcelona, Spain; ^3^ The National Institute of Complementary Medicine, Health Research Institute, Western Sydney University, Sydney, NSW, Australia

**Keywords:** cancer, digital health, electronic health, public health, implementation science, artificial intelligence, health equity

**Editorial on the Research Topic** Digital health quality, acceptability, and cost: steps to effective continuity of cancer care

Over 19 million people were diagnosed with cancer globally in 2020 (1). Recently, digital health interventions, including electronic medical/health records, telemonitoring, online patient portals, artificial intelligence (e.g., machine learning) and web-, mobile-, and text message-based interventions have become commonplace across the cancer care continuum, from cancer screening to post-treatment follow-up (2, 3). Benefits of digital health interventions include improved access to and delivery of cancer screening, decision aids, health information, management and tracking tools (e.g., smart watches, apps, websites), including psychosocial and physical health, for people living with and beyond cancer and their caregivers (2–8). Moreover, digital health interventions are scalable, adaptable and can be co-designed with multidisciplinary teams, including end-users, researchers, and clinicians, to address unmet healthcare needs (9). However, many barriers to access and use of digital health interventions exist, especially in low-resource areas and low-income countries (10, 11). Limited access to digital health technologies and limited technological skills or abilities to seek and understand health information from digital health sources (i.e., low digital health literacy) can lead to inequities in care delivery (12, 13). Moreover, receiving too much information from digital health sources can result in negative experiences, including fear (14). Sustainability of digital health interventions can also be problematic due to lack of resources (e.g., funding, workforce capacity) (15). The contributions in this Research Topic highlight the importance of inclusive co-design and equitable delivery of digital health interventions in cancer care.

Co-designing health innovations with people with diverse expertise (e.g., lived-experience, clinical, research) has been found to improve the quality of the innovation and users' perceived acceptability and utility (16). In this Research Topic, Morton et al. highlight how the design of a surgery decision aid for people with genetic predisposition of cancer was improved by involving multidisciplinary expertise in co-design. The original decision aid included detailed descriptions of the decision options (e.g., have surgery now or decide later), pros and cons of each decision and a quiz to indicate the most suitable decision. Using an iterative mixed-methods approach, participants made important alterations to the original decision aid, including a desire for concise descriptions of each decision option, including “do nothing”, and up-front implications of the decisions to set “realistic expectations”, with option to read additional information if desired. They also suggested a list of frequently asked questions, and a personally tailored summary of quiz results to facilitate clinician communications. Morton et al. emphasized the importance of including diverse co-designers from various jobs and ethnicities to facilitate development of future decision aids.

Once digital health interventions are designed, end-user testing is important for understanding acceptability, utility, and potential adaptations. Virtual patient platforms, including patient portals, are often used to support health self-management (2). In this Research Topic, Lamarche et al. describe how a fear of cancer recurrence program for patients was adapted into a program to support caregivers using a mixed methods, multidisciplinary approach, which was successfully user-tested by caregivers and therapists (17). Further, results from O’Connor et al. mixed methods evaluation (service use data, survey, interviews) of a patient portal to support follow-up care for 627 men with prostate cancer with low risk of recurrence revealed that within the portal, participants were most likely to access their test results and the communication systems (e.g., secured messaging, email) to contact their clinical team. Most participants felt the portal was quick, easy, convenient and time-saving compared to traveling to the hospital and reduced stress and facilitated communication with clinicians. However, people who declined to participate reported that digital equity was an issue, due to a lack of computer, internet, or technical skills. Participants suggested provision of technical support and technology could reduce barriers.

In low-income countries and low-resource areas, digital health inequities are exacerbated. For example, although there is some evidence of patients' acceptability and utility of virtual patient platforms (e.g., electronic personal health records; ePHRs), successful implementation is limited (17, 18). Wubante et al. conducted a cross sectional questionnaire of 402 health professionals in Ethiopia to evaluate their knowledge and attitudes regarding electronic personal health records. Most (93.5%) had never used ePHRs before but 64.4% perceived them to be useful for managing health and 55.5% had favorable attitudes, especially those with to the required technology, high digital health literacy and skills and access to computer training. Wubante et al. suggest providing training about technical aspects of ePHRs and their usefulness for health professionals could improve knowledge, attitudes, and use.

Digital health literacy plays a critical role in people's ability to use digital health innovations effectively. Nguyen et al. suggest that a validated measure of digital health literacy is key to understanding if a person is proficient enough to adopt a novel digital health intervention or require additional education or training. Nguyen et al. conducted an exploratory and confirmatory factor analysis to validate a novel digital health literacy tool for Vietnamese adolescents in Vietnam (*N* = 236). Results revealed that the tool was valid across gender, education, marital status, age, location, and household economy, which may facilitate collection of future high-quality digital health literacy data.

Throughout the cancer care continuum, routinely collected clinical data can also be evaluated using artificial intelligence, such as machine learning, to predict patient health outcomes. In a population-based retrospective cohort study (*N* = 52,199), Zhang et al. used machine learning to predict lymph node metastases in patients with renal cell carcinoma, based on their age, sex, tumour laterality, T and M stages, tumour size, histology, and grade. Their model showed high internal and external validity (AUC of 0.930 and 0.958, respectively) and good clinical applicability. As a result, Zhang et al. released a freely available online risk calculator.

This Research Topic *Digital Health Quality, Acceptability, and Cost: Steps to Effective Continuity of Cancer Care* provides readers with emerging evidence that furthers understanding of how digital health interventions can be harnessed to support patients, caregivers and healthcare professionals throughout the cancer care continuum and improve health outcomes. Equity, digital health literacy and sustainability are central considerations for successful digital health integration and usage in cancer care, which can be achieved through multidisciplinary codesign and testing.
